# Evaluation of contemporary echocardiography for the detection of cardiac sarcoidosis

**DOI:** 10.1186/s44156-026-00111-9

**Published:** 2026-04-27

**Authors:** Joseph Okafor, Alessia Azzu, Raheel Ahmed, Kshama Wechalekar, Athol Wells, A. John Baksi, Rakesh Sharma, Peter Collins, Nilesh Sutaria, Petros Nihoyannopolos, Roxy Senior, Vasileios Kouranos, Rajdeep S. Khattar

**Affiliations:** 1 Department of Echocardiography, Royal Brompton & Harefield Hospitals, Guy’s and St. Thomas’ NHS Foundation Trust, London, SW3 6NP UK; 2 National Heart & Lung Institute, Imperial College London, London, UK; 3 Cardiovascular Magnetic Resonance Unit, Royal Brompton & Harefield Hospitals, Guy’s and St. Thomas’ NHS Foundation Trust, London, UK; 4 Cardiac Sarcoidosis Service, Royal Brompton & Harefield Hospitals, Guy’s and St. Thomas’ NHS Foundation Trust, London, UK; 5 Department of Nuclear Medicine and PET, Royal Brompton & Harefield Hospitals, Guy’s and St. Thomas’ NHS Foundation Trust, London, UK

**Keywords:** Cardiac sarcoidosis, Echocardiography, Strain, Cardiac magnetic resonance, Nuclear cardiology

## Abstract

**Background:**

The ability of echocardiography (TTE) to diagnose cardiac sarcoidosis (CS) has traditionally been limited by its low sensitivity. We sought to determine the optimal TTE parameters to detect cardiac involvement in sarcoidos is with the inclusion of strain and 3DE to standard conventional TTE data.

**Methods:**

Consecutive patients referred for evaluation of suspected CS were prospectively recruited and underwent symptom evaluation, 12-lead ECG, ambulatory ECG monitoring, TTE with 3DE and strain, cardiac magnetic resonance and ^18^F-FDG-PET. We identified the TTE phenotype most likely to identify CS. We evaluated the ability of a novel multi-parametric approach to screen for cardiac involvement among sarcoidosis patients and compared this to existing criteria.

**Results:**

Of the 181 patients recruited (mean age 55 ± 11 years, 60% male, mean LVEF 57 ± 10%), 106 (59%) were diagnosed with CS. The strongest TTE parameters to predict CS were the presence of a dilated LV cavity (end-diastolic/end-systolic ≥ 71/28ml/m^2^ females; ≥80/32ml/m^2^ males), LVEF < 50%, RWMA involving multiple myocardial territories and basal septal thinning. This ‘probable CS’ TTE model had 96% specificity, 33% sensitivity and 92% PPV for the diagnosis of CS. The strain parameters most specific for a CS diagnosis were LVGLS and LVGCS greater than − 13% and − 15%, respectively. Adding strain or 3D data did not significantly improve the overall diagnostic ability of TTE. The optimal screening strategy for CS among sarcoidosis patients involved evaluation of symptoms, ECG or ambulatory ECG monitoring, and ‘probable CS’ TTE. This approach had a sensitivity of 90%, compared to 77% and 84% for the 2014 Heart Rhythm Society and 2020 American Thoracic Society criteria, respectively. In the asymptomatic patient, the absence of abnormal rhythm data and a normal TTE excluded CS in 94% of patients.

**Conclusion:**

The presence of LV dilatation, LV systolic impairment, multi-territory RWMA and basal septal thinning are the most specific TTE parameters for the detection of CS. In combination with symptom evaluation and ECG data, comprehensive echocardiography remains a useful screening tool among sarcoidosis patients with suspected cardiac involvement.

**Supplementary Information:**

The online version contains supplementary material available at 10.1186/s44156-026-00111-9.

## Introduction

Cardiac sarcoidosis (CS) is considered rare, with clinically manifest cardiac involvement occurring in 5% of systemic sarcoidosis patients. However, autopsy studies suggest a prevalence closer to 25–30% of sarcoidosis patients implying a greater burden of silent disease [1]. Establishing a diagnosis of CS is important as patients are at heightened risk of heart failure, conduction abnormalities, ventricular arrhythmias and sudden cardiac death [2, 3]. An increased awareness of the condition along with the development of cardiac magnetic resonance (CMR) imaging for the detection of fibrosis, and cardiac 18 F-flurodeoxyglucose positron emission tomography (FDG-PET) for myocardial inflammation, have led to higher detection rates of CS in recent years [4]. Echocardiography is used as a screening tool whereby the presence of regional wall motion abnormalities (RWMA), aneurysm formation or left ventricular dysfunction should prompt further investigation [5]. For diagnosis based on the 2014 Heart Rhythm Society (HRS) statement, the only criterion applicable to echocardiography is left ventricular ejection fraction (LVEF) < 40% [4]. Since most CS patients have a normal LVEF, the sensitivity of conventional echocardiography for detecting CS may be as low as 25–32% [6–8]. On this basis the 2020 American Thoracic Society (ATS) guidelines differ to the HRS in that the authors do not recommend TTE for the screening of sarcoidosis patients [9]. However, newer advances such as speckle-tracking echocardiography (STE) have emerged as a more sensitive technique for detecting early subclinical LV dysfunction. Three-dimensional echocardiography (3DE) including 3D STE has demonstrated incremental value in measuring LV volumes and biomechanics. Data on the incremental role of strain or 3DE in diagnosing CS is mainly limited to small retrospective studies evaluating a limited number of strain parameters [10–18].

We aimed to prospectively evaluate the ability of comprehensive echocardiography incorporating strain and 3D imaging to diagnose CS among a contemporary patient population. We sought to determine the echocardiographic phenotype most specific for the detection of CS. Additionally, we evaluated the optimal screening strategy for identifying cardiac involvement among sarcoidosis patients and compared this to current guidelines.

## Methods

### Patient population and cardiac workup

This was a prospective observational single-centre cohort study of consecutive patients aged ≥ 18 years referred to Royal Brompton Hospital, London, for evaluation of suspected CS between October 2021 and December 2023. As part of their cardiac workup, all patients underwent clinical assessment by a physician with specialist expertise in CS, 12-lead electrocardiogram (ECG) +/- ambulatory Holter monitoring, comprehensive transthoracic echocardiography (TTE), CMR imaging and cardiac FDG-PET. An abnormal ECG was defined as the presence of left or right bundle branch block, pathological Q-waves, sustained or non-sustained ventricular tachycardia (NSVT), ventricular ectopy (VE), any degree of atrioventricular (AV) block or supraventricular arrhythmia. An abnormal Holter was defined as the presence of VE burden > 10%, supraventricular ectopic burden > 10%, NSVT (≥ 3 consecutive ventricular beats ≥ 100 bpm for < 30s), sustained VT, supraventricular tachycardia, AV block or sinus node dysfunction (sinoatrial block, tachy-brady syndrome, sinus pauses, post-conversion asystole).

Participants were excluded if they had significant structural heart disease including severe coronary artery disease, at least moderate valvular disease and known congenital heart disease. Severe coronary artery disease was defined as previous coronary artery bypass grafting, previous ST-elevated myocardial infarction (STEMI), or known coronary stenosis ≥ 75% in one or more coronary arteries. Participants who could not undergo a CMR scan or were unable to provide written informed consent were also excluded. The study was approved by the Research Ethics committee of the Health Research Authority, United Kingdom (REC 22/PR/0459).

### Diagnosis of systemic and cardiac sarcoidosis

The diagnosis of systemic sarcoidosis was based on a compatible clinical picture with radiological evidence or histological confirmation of non-caseating granuloma. In all patients, the final diagnosis of CS was made following a detailed multidisciplinary team (MDT) discussion with specialist review of all clinical and imaging data. The MDT decision was guided by the HRS criteria [4]. In the absence of histological evidence of extracardiac sarcoidosis, the 2016 Japanese Circulation Society (JCS) criteria were used [19] (Supplemental 1). In the absence of histological or radiological evidence of extracardiac sarcoidosis, a diagnosis of isolated CS was ascribed to those with a strong clinical suspicion of CS, following CMR and whole-body FDG-PET imaging, in line with 2016 JCS criteria [19]. Those with clear alternative diagnoses identified during MDT discussion were excluded from the final analysis. Our screening strategy was compared to the HRS and American Thoracic Society (ATS) screening recommendations (Supplemental 2) [4, 19].

### Two-dimensional echocardiographic protocol

2DE was performed using a Philips EPIQ ultrasound system (Philips Medical Systems, Eindhoven, Netherlands) by experienced echocardiographers. Standard views were obtained and all measurements were recorded based on current British Society of Echocardiography (BSE) recommendations [20, 21]. LVEF was calculated using Simpson’s biplane method. Impaired LVEF was defined as LVEF < 50%. Increased LV dimension was defined as internal diameter in diastole/systole of ≥ 5.7/4.2 cm for males and ≥ 5.2/3.8 cm for females. Increased LV volume was defined as indexed end-diastolic (EDV) and/or end-systolic volume (ESV) ≥ 80/32ml/m^2^ for males and ≥ 71/28ml/m^2^ for females. Left ventricular hypertrophy (LVH) was defined as septal wall thickness ≥ 13 mm in males and ≥ 12 mm in females. Left ventricular mass index (LVMi) was derived by the Devereux method. Elevated LVMi was defined as ≥ 111 g/m^2^ in males and ≥ 100 g/m^2^ in females. Dilated LA diameter and volume were defined as ≥ 4.0 cm and ≥ 35ml/m^2^, respectively. Presence of diastolic dysfunction was determined as per current BSE guidelines [22]. Presence of RWMA within each myocardial territory was determined visually in the standard echocardiographic views. Multi-territory RWMA was defined as RWMA in ≥ 2 myocardial regions (septal, anterior, anterolateral, inferolateral, inferior, apical). Each of the 17 segments were assigned a score (1 = normal, 2 = hypokinesis, 3 = akinesis, 4 = dyskinesis and 5 = aneurysmal). Wall motion score index (WMSi) was calculated by dividing the sum of the scores by the number of segments. Basal septal thinning was defined as basal interventricular septal (IVS) thickness ≤ 4 mm measured 10 mm from the aortic annulus, and/or a basal IVS/mid-IVS ratio ≤ 0.6 [23].

### 2DE speckle-tracking strain analysis

Standard 2D images of the LV were obtained in the parasternal short-axis (apical, mid-papillary, basal) views and the apical 2-, 3- and 4-chamber views. A dedicated right ventricular (RV) focussed 4-chamber view was obtained. Dedicated views of the left atrium (LA) were used with care taken not to foreshorten the LA chamber. Images were recorded with frame rates between 50 and 90 frames per second (fps) to ensure optimal software analysis. Offline speckle tracking analysis of global longitudinal strain (GLS), RV and LA strain were performed using AutoStrain LV/RV/LA software (TomTec Imaging Systems). Global circumferential strain (GCS) analysis was performed using 2D CPA (TomTec Imaging Systems). Endocardial border tracking was achieved automatically using 2 points in the annular region and 1 point in the apical segments. Tracking quality was visually verified and segments that failed initial tracking were manually adjusted. Segments that could not be tracked properly after manual adjustment were rejected. If ≥ 3 LV or ≥ 2 RV segments were inadequate, the patient’s strain data was excluded from the final analysis. The following 2D endocardial STE values were obtained: peak-systolic LVGLS, LVGCS, RV free wall strain (RVFWS), RVGLS and end-systolic LA reservoir, conduit and contractile strain. LVGLS and GCS were obtained by averaging values from the 3 apical or parasternal short-axis views using a 17-segment model. RVFWS was obtained by averaging the free wall basal, mid and apical segment values. RVGLS was measured from the average values of the RV free wall and RV septal wall. The cut-off for mildly impaired global strain values was based on large population meta-analyses data as follows: LVGLS − 17%, LVGCS − 21%, RVFWS − 18% RVGLS − 16%, LA reservoir 39%, LA conduit − 23% and LA contractile − 17% [24–26].

### Three-dimensional echocardiographic protocol

3DE full-volume single and multibeat datasets were obtained on a Philips EPIQ ultrasound system with a X5-1 matrix array transducer (frequency of 5 − 1 MHz) using the tissue harmonic mode with an ECG-gated acquisition. Images were acquired based on current recommendations [27]. Offline vendor-independent software 4D RV-Function and 4D LV-Analysis (both TomTec Imaging Systems GmbH, Unterschleissheim, Germany) were used to calculate RV and LV volumes, respectively. After alignment of 3D datasets and identification of key landmarks, EDVs and ESVs were estimated with the use of semi-automated frame-by-frame endocardial contours. These were corrected manually when necessary. The papillary muscles and trabeculae were included in cavity evaluation when tracing chamber volumes. In the case of poor image quality due to ultrasound dropout, data was excluded from the final analysis. Image quality was adjudged poor if one-half of the RV in the coronal view or ≥ 3 adjacent segments in the LV were affected. Impaired 3D LVEF and RVEF were defined as EF < 50% and < 45%, respectively. LV cavity dilatation was defined as EDV and/or ESV ≥ 80/33ml/m^2^ for males and ≥ 73/28ml/m^2^ for females [28]. RV cavity dilatation was defined as EDV and/or ESV ≥ 96/44ml/m^2^ for males and ≥ 82/37ml/m^2^ for females [29].

### 3DE speckle-tracking strain analysis of the LV

3D speckle-tracking analysis was performed offline using 4D LV-Analysis (TomTec Imaging Systems). The region of interest was set to cover the full thickness of the LV myocardium in all segments. The software provided the following peak-systolic 3D strain components: 3D GLS, 3D GCS, 3D GRS, 3D global principal strain (GPS), 3D Twist and 3D Torsion. LV twist (°) was measured as the net difference between the basal and apical rotation angles (LV twist = apical LV rotation–basal LV rotation). LV torsion was calculated as the net LV twist normalized with respect to LV end-diastolic longitudinal length between the LV apex and the mitral plane (LV torsion (°/cm) = LV twist /end-diastolic length).

### CMR & FDG-PET protocol

The CMR and FDG-PET protocols are detailed in Supplemental 3.

### Statistical analysis

Continuous variables were expressed as the mean ± standard deviation when normally distributed or median with interquartile range (IQR). Shapiro-Wilks testing was used to determine normality. Categorical variables were presented as frequency with corresponding percentages. Differences in continuous variables between 2 groups were tested using Student’s t test or Mann-Whitney *U* tests accordingly. Categorical variables between 2 groups were compared using χ2 test. To define the echocardiographic models most likely to detect CS we first identified the best echocardiographic predictors as clinically useful binary variables. Guideline-derived cut-off values for established parameters 2D and 3D LVEF, LV cavity dimension, 2D and 3D LV cavity volume, LVMi, 3D RVEF, 3D RV cavity volume, LA diameter and LA volume were used as described in the methods section. The cut-off for mildly impaired global strain values was as described in the methods. The cut-off for severely impaired global strain values represented the ≥ 90th percentile of impaired strain values across our cohort. For other continuous variables, receiver operating characteristic (ROC) analysis was used to determine the area under the curve (AUC) for parameters to predict CS and the optimal cut-off was identified by the Youden Index. The diagnostic ability of each parameter was assessed by identifying the sensitivity, specificity, positive likelihood ratio (LR+), negative likelihood ratio (LR-), positive predictive value (PPV) and negative predictive value (NPV) of that parameter to detect CS. The gold standard was a diagnosis of CS as provided by the MDT. Finally, three echocardiography models, graded by degree of specificity, were designed: (i) *probable CS* – including parameters with ≥ 99% specificity, (ii) *highly likely CS* – including parameters with 95–98% specificity and (iii) *moderately likely CS* – including parameters with 90–94% specificity. The diagnostic ability of 3 models was examined both across the whole cohort and purely among those with extracardiac sarcoidosis. To assess intra-observer variability for STE parameters, the same reader re-evaluated images from 30 random patients. Reliability was determined by intraclass correlation coefficient (ICC) using a two-way mixed model for absolute agreement. For all tests, a 2-tailed *P* value of < 0.05 was considered as statistically significant. Statistical analyses were conducted using Stata software version 18.0 (StataCorp, College Station, Texas, USA).

## Results

### Patient characteristics

**Fig. 1:**
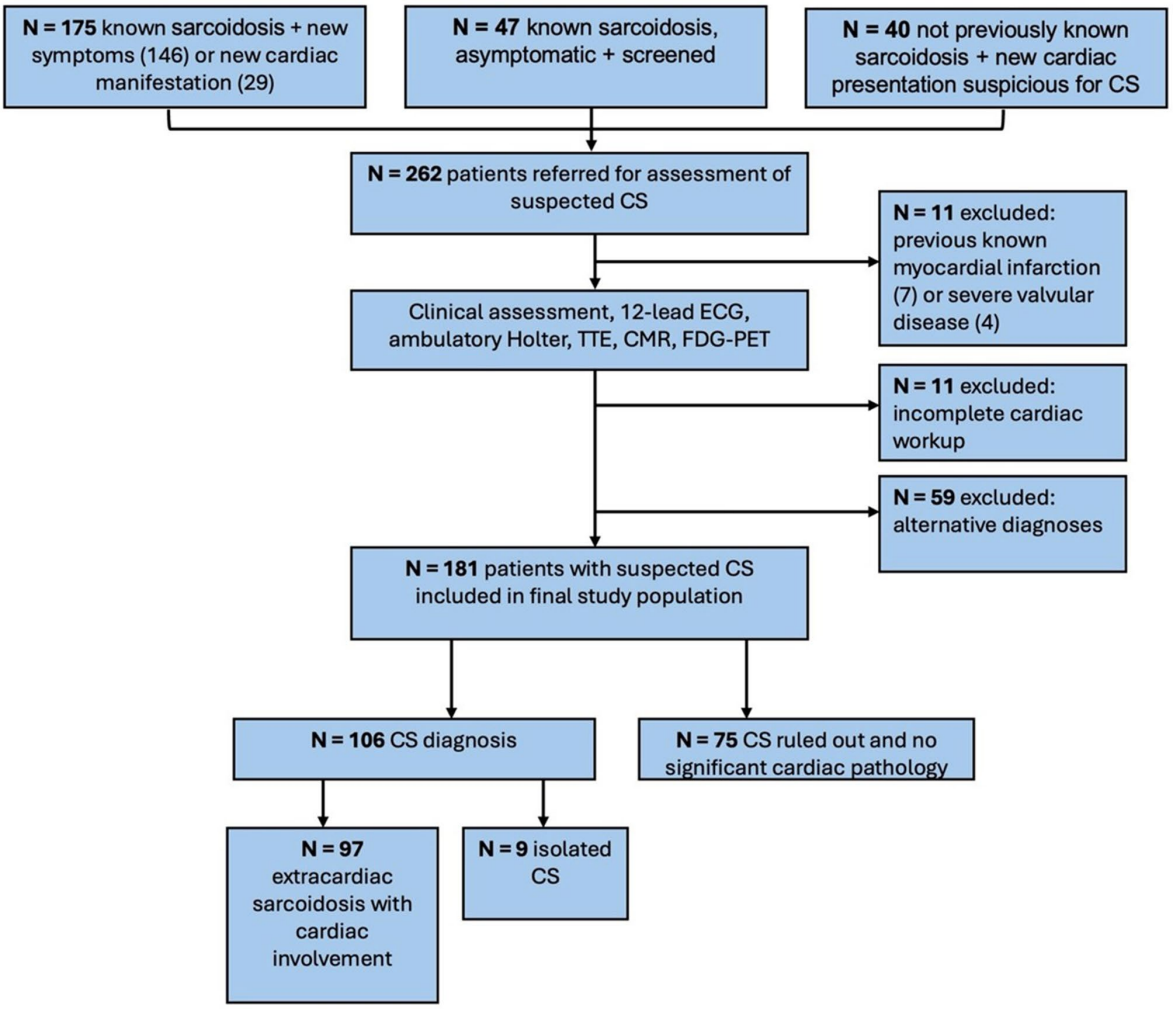
Overview of patient pathway from mode of presentation to diagnosis. CMR = cardiovascular magnetic resonance imaging; CS = cardiac sarcoidosis; FDG-PET = 18 F-flurodeoxyglucose positron emission tomography; TTE = transthoracic echocardiography

Of the 262 patients recruited, 11 were excluded due to incomplete data, 7 had previous MI, and 4 severe valvular disease. Of the remaining 240 patients who underwent full cardiac workup, 59 had alternative diagnoses and were also excluded (Fig. 1**)**. The final study population consisted of 181 patients, of whom 167 (92%) had extracardiac sarcoidosis. Among those with extracardiac involvement, 140 (77%) had biopsy-proven extracardiac sarcoidosis and 27 (23%) had radiological evidence of pulmonary sarcoidosis. Following MDT discussion, a diagnosis of CS was made in 106 (56%) patients of whom 79 (75%) fulfilled the HRS clinical criteria and 71 (67%) fulfilled the JCS criteria. All CS patients fulfilled at least one of the two criteria and 44 (42%) fulfilled both. Nine patients who met the JCS criteria had isolated CS.

Among the whole group, the HRS criteria had 75% sensitivity, 99% specificity, 98% PPV, and 83% NPV for diagnosing CS with a positive and negative likelihood ratio of 49.93 (95% CI 12.56-198.49) and 0.26 (0.19–0.36), respectively. The JCS criteria had 67% sensitivity, 99% specificity, 99% PPV and 79% NPV with positive and negative likelihood ratios of 89.75 (12.68-635.42) and 0.33 (0.25–0.44), respectively. While the HRS criteria could only be applied to the 140 patients with biopsy-proven extracardiac sarcoidosis, the JCS criteria were applicable to all 181 study patients, which accounted for the greater positive likelihood ratio.

### Baseline demographics and presenting symptoms

Among the study population, the mean age was 54 ± 11 years, 59% were male, 75% were Caucasian, 14% were Asian and 11% were Black (Table 1). There was a history of smoking in 19%, hypertension in 24%, and diabetes in 9%. There was no significant difference in demographic data between CS + and CS- patients. The majority (90%) of patients had pulmonary sarcoidosis, while 50% had mediastinal lymph nodal, 10% had cutaneous, 9% ophthalmic, 7% renal, 4% hepatobiliary, 4% neurological, 4% musculoskeletal, and 3% splenic involvement. The prevalence of pulmonary hypertension was low (1%). Patients presented with cardiac-sounding symptoms in 72% of cases including palpitations (39%), chest pain (25%), and pre-syncope/syncope (19%). A greater proportion of CS+ patients presented with pre-syncope or syncope (*p* = 0.006) or a cardiac manifestation of high-grade AV block (*p* < 0.001), ventricular arrhythmia (*p* = 0.006), or systolic heart failure (*p* = 0.001).

**Table 1:** Baseline demographics of the whole group and comparisons between those with and without cardiac involvement

	Whole group(*n* = 181)	CS +(*n* = 106)	CS –(*n* = 75)	*P value*
Age, years	54 ± 11	55 ± 11	53 ± 11	0.408
Male, n (%)	107 (59)	69 (65)	38 (51)	0.052
Ethnicity				
- Caucasian	136 (75)	81 (76)	55 (73)	0.190
- Black	20 (11)	14 (13)	6 (8)	
- Asian	25 (14)	11 (10)	14 (19)	
BMI, kg/m^2^	29.3 ± 6.1	29.6 ± 6.3	28.9 ± 5.8	0.484
Smoking history, n (%)	35 (19)	17 (16)	18 (24)	0.182
Hypertension, n (%)	43 (24)	25 (24)	18 (24)	0.948
Diabetes mellitus	17 (9)	11 (10)	6 (8)	0.589
Dyslipidemia, n (%)	19 (10)	10 (9)	8 (11)	0.785
CKD, n (%)	6 (3)	3 (3)	3 (4)	0.665
Pulmonary hypertension, n (%)	2 (1)	2 (2)	0	0.238
Presenting symptoms, n (%)	131 (72)	78 (74)	53 (71)	0.665
- Chest pain	45 (25)	23 (22)	22 (29)	0.242
- Palpitations	71 (39)	36 (34)	35 (47)	0.085
- Pre/syncope	34 (19)	27 (25)	7 (9)	**0.006**
Cardiac Manifestations				
- High-grade AV block	24 (13)	22 (21)	2 (3)	**< 0.001**
- VT/VF	10 (6)	10 (9)	0	**0.006**
- Systolic HF	19 (11)	18 (17)	1 (1)	**0.001**
Extra-cardiac sarcoidosis, n (%)	167 (92)	97 (92)	70 (93)	0.651
ECS biopsy, n (%)	140 (77)	79 (75)	61 (81)	0.281
Organ involvement, n (%)				
- Pulmonary	163 (90)	93 (88)	70 (93)	0.215
- Mediastinal LN	91 (50)	59 (56)	32 (43)	0.085
- Neurological	7 (4)	6 (6)	1 (1)	0.137
- Ophthalmic	17 (9)	9 (8)	8 (11)	0.621
- Skin	18 (10)	13 (12)	5 (7)	0.215
- MSK	7 (4)	5 (5)	2 (3)	0.481
- Renal	12 (7)	6 (6)	6 (8)	0.533
- Liver/Biliary	7 (4)	5 (5)	2 (3)	0.481
- Spleen	5 (3)	3 (3)	2 (3)	0.947

Bold figures indicate statistical significance (*p* < 0.05)

### Investigations and treatment at time of presentation

Pulmonary function test results did not differ significantly between CS + and CS- patients (Table 2). However, CS+ patients had more abnormal 12-lead ECGs or ambulatory Holter monitors. On CMR, a greater proportion of CS+ patients had a positive T2-STIR signal (18% vs. 1%, *p* < 0.001) and LGE (98% vs. 21%, *p* < 0.001). Among those considered not to have CS, 16 (21%) patients had LGE in a non-specific distribution. As per the diagnostic criteria, CS+ patients had more abnormal FDG/Rb-82-PET (70% vs. 8%, *p* < 0.001) including more FDG uptake, perfusion defects and metabolism-perfusion mismatch patterns.

Before investigation, 51% of patients were on immunosuppressive therapy for extracardiac sarcoidosis consisting of steroids in 43% of patients with the remainder on steroid-sparing agents. At the time of diagnosis, prior use of immunosuppressive therapy was similar across the two groups, but a higher proportion of CS+ patients were being treated with beta-blockers or ACE-i/ARBs. Prior insertion of implantable cardioverter defibrillator (ICD) and cardiac resynchronization therapy-defibrillators (CRT-D) were greater among CS+ patients.

**Table 2:** Investigations and treatment of the whole group and comparisons between those with and without cardiac involvement

	Whole group(*n* = 181)	CS +(*n* = 106)	CS –(*n* = 75)	*P value*
Abnormal 12-lead ECG, *n* (%)	58 (32)	49 (46)	9 (12)	**< 0.001**
Abnormal Holter, n (%)	43 (24)	36 (34)	7 (10)	**< 0.001**
LGE on CMR, n (%)	120 (66)	104 (98)	16 (21)	**< 0.001**
Abnormal FDG/Rb-82 PET, n (%)	80 (44)	74 (70)	6 (8)	**< 0.001**
FDG uptake, n (%)	54 (30)	52 (49)	2 (3)	**< 0.001**
Perfusion defect, n (%)	61 (34)	55 (52)	6 (8)	**< 0.001**
Metabolic mismatch pattern, n (%)	34 (19)	32 (30)	2 (3)	**< 0.001**
Lung function testing (% predicted)				
- FEV1	88 ± 21	87 ± 21	91 ± 20	0.326
- FVC	98 ± 21	98 ± 20	99 ± 22	0.790
- TLCo	73 ± 18	73 ± 18	72 ± 18	0.859
Any immunosuppression, n (%)	92 (51)	51 (48)	41 (55)	0.385
Corticosteroid	77 (43)	41 (39)	36 (48)	0.212
Steroid sparing agent	48 (25)	27 (15)	18 (24)	
Beta-blocker, n (%)	43 (24)	36 (34)	7 (9)	**< 0.001**
ACE-inhibitor/ARB, n (%)	54 (30)	42 (40)	12 (16)	**0.001**
Permanent pacemaker	8 (4)	6 (6)	2 (3)	0.334
Implantable cardioverter defibrillator	14 (8)	13 (12)	1 (1)	**0.007**
Cardiac resynchronization therapy	7 (4)	7 (7)	0	**0.023**

Bold figures indicate statistical significance (*p* < 0.05)

### Conventional two-dimensional echocardiographic parameters and CS diagnosis

On 2DE, LVESD, LVEDD, IVSd, LVMi, LVEDVi, LVESVi, diastolic dysfunction, LA diameter, LAVi and RV basal diameter were all significantly greater in CS+ compared to CS- patients (Table 3). A dilated LV cavity volume was noted in 29% of CS+ patients compared to 3% in CS- patients (*p* < 0.001). The CS+ group had lower LVEF (56% ± 11% vs. 62% ± 5%, *p* < 0.001) and a higher proportion of patients with LVEF < 50% (*p* < 0.001). CS+ patients were more likely to have an RWMA (32% vs. 4%, *p* < 0.001), multi-territory RWMA (21% vs. 1%, *p* < 0.001), and basal septal thinning (13% vs. 0%; *p* = 0.001). Correspondingly, WMSi was significantly higher in the CS+ group (1.24 ± 0.45 vs. 1.02 ± 0.25, respectively; *p* < 0.001).

**Table 3:** Two-dimensional echocardiographic parameters and comparisons between those with and without cardiac sarcoidosis

	Whole group(*n* = 181)	CS +(*n* = 106)	CS –(*n* = 75)	*P value*
**LV dimensions & systolic function**				
LVESD, cm	4.9 ± 0.7	5.0 ± 0.8	4.5 ± 0.6	**< 0.001**
LVEDD, cm	3.3 ± 0.8	3.5 ± 0.9	3.0 ± 0.6	**< 0.001**
IVS(d), cm	0.9 ± 0.2	0.9 ± 0.2	0.8 ± 0.2	**0.026**
LVMi, g/m^2^	77 ± 22	82 ± 22	70 ± 12	**< 0.001**
LVEDVi, ml/m^2^	49 (41–59)	54 (45–69)	43 (37–52)	**< 0.001**
LVESVi, ml/m^2^	20 (15–25)	22 (18–30)	16 (14–20)	**< 0.001**
Increased LV dimension, n (%)	38 (21%)	31 (29%)	7 (9%)	**0.001**
Increased LV volume, n (%)	33 (18%)	31 (29%)	2 (3%)	**< 0.001**
LVEF, %	58 ± 9	56 ± 11	62 ± 5	**< 0.001**
LVEF < 50%, n (%)	22 (12%)	21 (20%)	1 (1%)	**< 0.001**
**LV Diastolic function**				
Diastolic dysfunction, n (%)	28 (15%)	24 (23%)	4 (5%)	**0.002**
LA diameter, cm	3.5 ± 0.7	3.6 ± 0.7	3.3 ± 0.6	**0.007**
LA volume index, ml/m^2^	26 ± 10	28 ± 10	24 ± 11	**0.033**
Dilated LA (LAVi ≥ 35ml/m^2^), n (%)	21 (12%)	16 (15%)	5 (7%)	0.081
**Right heart assessment**				
RV basal diameter, cm	3.7 ± 0.6	3.8 ± 0.6	3.5 ± 0.6	**0.004**
RV S’, cm/s	13.0 ± 2.6	12.9 ± 2.7	13.0 ± 2.4	0.788
RV FAC, %	43 ± 9	42 ± 9	44 ± 8	0.199
TAPSE, cm	2.2 ± 0.4	2.2 ± 0.4	2.2 ± 0.4	0.244
PASP, mmHg	23 ± 9	24 ± 9	22 ± 8	0.115
PV acceleration time, ms	126 ± 30	123 ± 31	131 ± 29	0.072
RVOT VTI: PASP ratio	0.77 ± 0.34	0.75 ± 0.37	0.80 ± 0.30	0.332
**Wall motion abnormality**				
Single territory RWMA, n (%)	14 (8%)	12 (11%)	2 (3%)	**0.032**
Multi-territory RWMA, n (%)	23 (13%)	22 (21%)	1 (1%)	**< 0.001**
Basal septal thinning, n (%)	14 (8%)	14 (13%)	0	**0.001**
Wall motion score index	1.15 ± 0.37	1.24 ± 0.45	1.02 ± 0.12	**< 0.001**
Septal RWMA, n (%)	28 (15%)	27 (25%)	1 (1%)	**< 0.001**
Anterolateral RWMA, n (%)	11 (6%)	11 (10%)	0	**0.004**
Anterior RWMA, n (%)	12 (7%)	12 (11%)	0	**0.003**
Inferior RWMA, n (%)	23 (13%)	22 (21%)	1 (1%)	**< 0.001**
Inferolateral RWMA, n (%)	15 (8%)	15 (14%)	0	**0.001**
Apical RWMA, n (%)	10 (6%)	8 (8%)	2 (3%)	0.157
RV free wall RWMA, n (%)	9 (5%)	7 (7%)	2 (3%)	0.230

Bold figures indicate statistical significance (*p* < 0.05)

### Three-dimensional echocardiographic parameters and CS diagnosis

Three-dimensional echocardiography was feasible in 91/181 (50%) patients with the others limited by poor endocardial definition, limited acoustic windows or arrhythmia. LVEDVi, LVESVi, RVEDVi and RVESVi were significantly greater in the CS+ group while LVEF and RVEF were lower (Table 4). Mean LVEF was lower by 1% on 3DE compared to 2DE (57 ± 8% vs. 58 ± 8%, *p* = 0.015) with 3DE identifying a greater proportion of CS patients with impaired LVEF < 50%.

**Table 4:** Three-dimensional echocardiographic parameters and comparisons between those with and without cardiac involvement

	Whole group	CS +	CS –	***P*** ** value**
**Left ventricle**	**(*n*** **= 91)**	**(*n*** **= 51)**	**(*n*** **= 40)**	
LVEDVi, ml/m^2^	59 (46–70)	65 (51–72)	55 (42–64)	**0.009**
LVESVi, ml/m^2^	25 (19–30)	25 (21–34)	23 (18–28)	**0.020**
Dilated LV cavity, n (%)	21 (23%)	16 (31%)	5 (13%)	**0.034**
LVEF, %	57 ± 8	55 ± 9	59 ± 5	**0.004**
LVEF < 50%, n (%)	13 (14%)	13 (25%)	0	**0.001**
LVEF < 40%, n (%)	3 (3%)	3 (6%)	0	0.119
**Right ventricle**	**(*n*** **= 68)**	***(n*** **= 38)**	**(*n*** **=** **30)**	
RVEDVi, ml/m^2^	57 (43–71)	63 (50–75)	53 (39–60)	**0.005**
RVESVi, ml/m^2^	28 (22–36)	32 (24–46)	24 (17–31)	**0.004**
Dilated RV cavity, n (%)	13 (19%)	11 (29%)	2 (7%)	**0.020**
RVEF, %	49 ± 7	47 ± 7	52 ± 7	**0.002**
RVEF < 45%, n (%)	16 (24%)	12 (32%)	4 (13%)	0.078

Bold figures indicate statistical significance (*p* < 0.05)

### Myocardial strain parameters and CS diagnosis

Two-dimensional STE assessment of LVGCS was feasible in 92%, LVGLS in 78%, RV strain in 77% and LA strain in 83% of ECS patients while 3D STE was feasible in 50%. Across the 2D strain parameters, LVGLS, LVGCS, RVFWS, RVGLS, LA reservoir and LA conduit strain were significantly more impaired in CS+ compared to CS- (Table 5). There was no significant difference in 3D STE values between the two groups. ICCs for global LV strain parameters are shown in Supplementary 4. Reproducibility was better for the 2D strain (LVGLS 0.911, 95% CI 0.781–0.962; LVGCS 0.960, 95% CI 0.903–0.981) compared to the corresponding 3D strain (LVGLS 0.763, 95% CI 0.559–0.879; LVGCS 0.746, 95% CI 0.532–0.871) measurements.

**Table 5:** Myocardial strain parameters and comparisons between those with and without cardiac sarcoidosis

	Whole group	CS +	CS –	*P value*
**2D Left ventricle**				
2D LVGLS, %	-18.4 ± 3.7	-17.3 ± 3.7	-19.9 ± 3.2	**< 0.001**
2D LVGCS, %	-22.6 ± 5.7	-21.5 ± 6.1	-24.2 ± 4.8	**0.002**
**3D Left ventricle**				
3D LVGLS, %	-19.3 ± 5.7	-18.3 ± 6.5	-20.6 ± 4.3	0.059
3D LVGCS, %	-27.8 ± 6.1	-27.1 ± 6.8	-28.6 ± 5.1	0.231
3D LVGRS, %	38.8 ± 11.5	36.5 ± 14.4	41.5 ± 6.0	0.044
3D LVGPS, %	-33.5 ± 6.4	-32.6 ± 7.6	-34.4 ± 4.5	0.201
3D Twist (°)	12.8 ± 6.6	12.3 ± 6.8	13.3 ± 6.4	0.509
3D Torsion (°/cm)	1.48 ± 0.79	1.37 ± 0.81	1.60 ± 0.76	0.181
**2D Right ventricle**				
RVFWS	-21.9 ± 5.6	-20.7 ± 5.8	-23.5 ± 5.1	**0.004**
RV4CS	-18.0 ± 4.6	-16.7 ± 4.6	-19.8 ± 4.0	**< 0.001**
**2D Left atrium**				
LA reservoir	34.1 ± 12.3	31.8 ± 11.8	37.3 ± 12.4	**0.007**
LA conduit	-18.8 ± 8.8	-17.4 ± 8.8	-20.8 ± 8.6	**0.022**
LA contractile	-15.8 ± 8.8	-15.2 ± 9.2	-16.6 ± 8.2	0.349

Bold figures indicate statistical significance (*p* < 0.05)

### Diagnostic value of individual echocardiographic parameters

The sensitivity, specificity, positive and negative predictive values for the key echocardiographic parameters are shown in Table 6. Severely impaired strain values represented those ≥ 90th percentile across the cohort. For LVGLS this was − 13%, LVGCS − 15%, RVFWS − 15%, RVGLS − 12% and LA reservoir strain 17%. Among the strain parameters LVGLS greater than − 13% had the highest specificity (91%) for a CS diagnosis.

**Table 6:** Diagnostic ability of TTE parameters among whole group

	Cut-off (where relevant)	Sens(95% CI)	Spec(95% CI)	PPV(95% CI)	NPV(95% CI)
**Chamber size and function**					
Dilated LV dimensions	≥ 5.7/4.2 cm (M) ≥ 5.2/3.8 cm (F)	29 (21–39)	91 (82–96)	82 (66–92)	48 (39–56)
Dilated LV volumes (indexed)	≥ 80/32ml/m^2^ (M) ≥ 71/28ml/m^2^ (F)	24 (16–33)	99 (93–100)	96 (80–100)	48 (40–56)
Increased septal wall thickness	≥ 13 mm (M)/ ≥12 mm (F)	12 (7–20)	95 (87–99)	77 (50–93)	43 (36–51)
Increased LV mass	≥ 111 g/m^2^ (M)/ ≥100 g/m^2^ (F)	12 (7–20)	93 (85–98)	72 (47–90)	43 (35–51)
LVEF < 50%		20 (13–29)	99 (93–100)	95 (77–100)	47 (39–55)
Diastolic dysfunction		23 (15–32)	95 (87–99)	86 (67–96)	46 (38–55)
Dilated LA diameter	≥ 4.0 cm	23 (15–32)	88 (78–95)	73 (55–87)	45 (36–53)
Dilated LA volume (indexed)	≥35ml/m^2^	20 (13–28)	91 (82–96)	75 (55–89)	44 (36–53)
Increased RV basal diameter	≥ 4.2 cm*	25 (17–34	91 (82–96)	79 (61–91)	46 (38–55)
**Wall motion abnormality**					
Single territory RWMA		11 (6–19)	97 (91–100)	86 (57–98)	44 (36–52)
Multi territory RWMA		21 (14–30)	99 (93–100)	96 (78–100)	47 (39–55)
Basal septal thinning		13 (7–21)	100 (95–100)	100 (77–100)	45 (37–53)
**3D Echo**					
Dilated LV volumes (indexed)	≥ 80/33ml/m^2^ (M) ≥ 72/29ml/m^2^ (F)	25 (14–38)	79 (63–90)	62 (38–82)	43 (31–55)
Dilated RV volumes (indexed)	≥ 88/45ml/m^2^ (M) ≥75/37ml/m^2^ (F)	16 (7–31)	76 (55–91)	54 (25–81)	35 (22–49)
LVEF < 50%		15 (7–28)	87 (72–96)	62 (32–86)	42 (31–54)
RVEF < 45%		26 (14–41)	80 (59–93)	69 (41–89)	39 (25–53)
**Impaired 2D strain**					
LVGLS					
Mild^1^	> -17%	32 (22–43)	75 (62–86)	66 (49–80)	43 (33–53)
Severe^2^	> -13%	11 (5–19)	91 (81–97)	64 (35–87)	41 (32–50)
LVGCS					
Mild	> -21%	36 (27–47)	60 (48–71)	55 (42–67)	41 (32–51)
Severe	> -15%	10 (5–18)	90 (79–95)	56 (31–79)	42 (34–51)
RVFWS					
Mild	> -18%	22 (13–32)	77 (64–87)	58 (39–76)	40 (31–50)
Severe	> -15%	11 (5–20)	88 (76–95)	56 (30–80)	40 (31–49)
RVGLS					
Mild	> -16%	35 (25–46)	78 (65–88)	71 (55–84)	44 (34–55)
Severe	> -12%	11 (5–20)	89 (78–96)	60 (32–84)	40 (31–49)
LA reservoir strain					
Mild	< 39%	68 (57–77)	36 (24–49)	59 (49–69)	45 (31–60)
Severe	< 17%	9 (4–17)	89 (79–96)	53 (27–79)	42 (34–51)

1. Mildly impaired strain values represent values below the guideline-defined lower limit of normal

2. Severely impaired strain values represent the ≥ 90th percentile of strain values across the cohort

*Indicates optimal cut-off determined by ROC analysis

### Echocardiography models and likelihood of CS detection

**Fig. 2:**
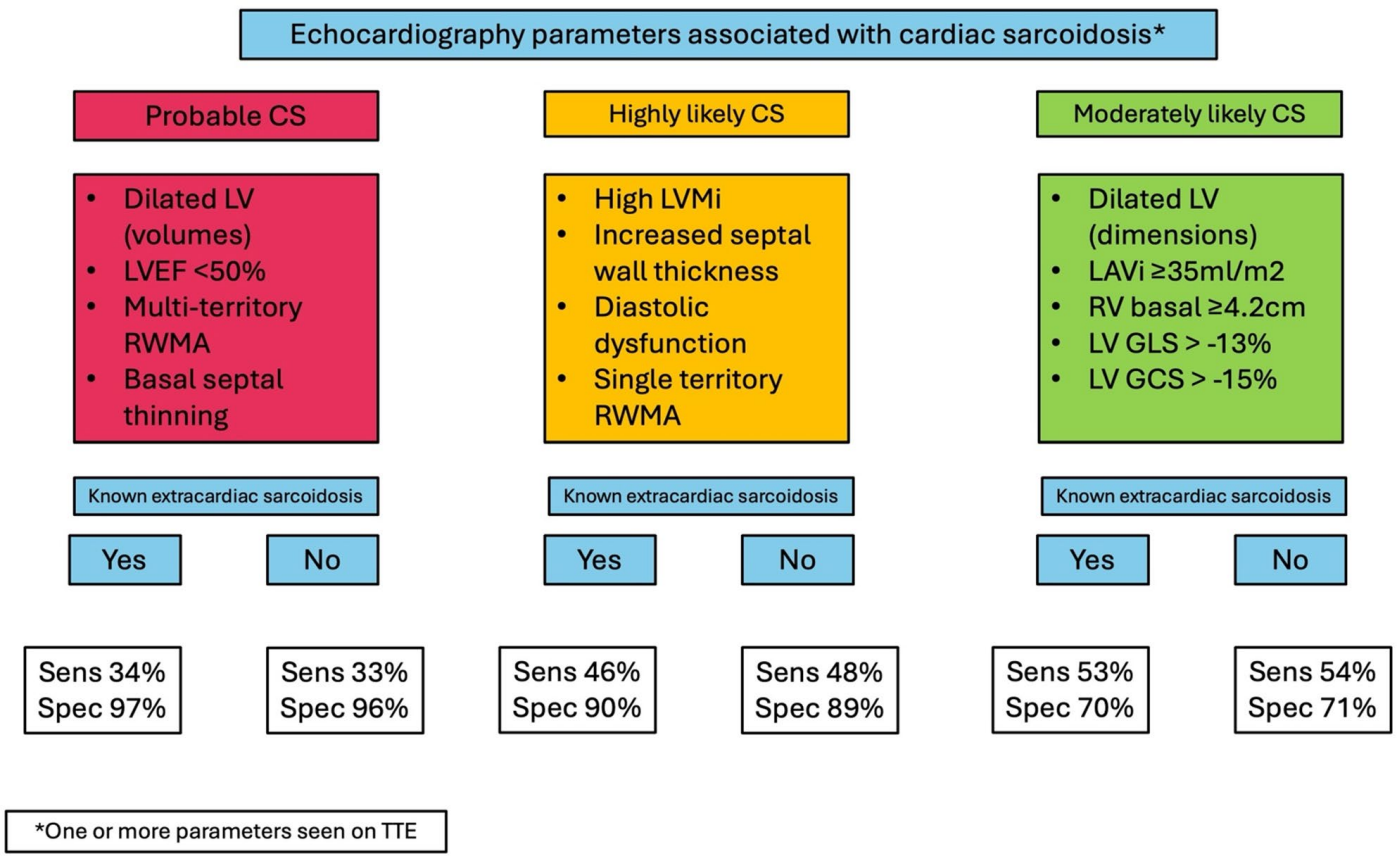
Transthoracic echocardiography (TTE) models graded by specificity for a cardiac sarcoidosis (CS) diagnosis across the whole cohort. One or more of the parameters from each group are identified on a TTE scan in order to grade that scan based on likelihood of a diagnosis of CS following onward CMR and FDG-PET. Probable CS TTE included parameters that individually carried specificity ≥ 99%. Highly likely CS TTE included parameters that individually carried specificity 95–98%. Moderately likely CS TTE included parameters that individually carried specificity 90–94%

Among our population, detection of CS was (Fig. 2):

(i) **Probable** if the echocardiogram showed any of the following: increased indexed LV volume, LVEF < 50%, multi-territory RWMA or basal septal thinning.
(ii) **Highly likely** if the echocardiogram showed any of the following: increased LV mass, septal hypertrophy, diastolic dysfunction, or RWMA involving a single territory.
(iii) **Moderately likely** if the echocardiogram showed any of the following: increased LV cavity dimension, LA volume dilatation, increased RV basal diameter, severely impaired LVGLS − 13%, or severely impaired LVGCS − 15%.

Table 7 shows the diagnostic ability of all three echocardiographic probability models among the whole population and those exclusively with extracardiac sarcoidosis. Among the whole cohort, the presence of a probable CS echocardiogram had 96% specificity, 92% PPV and positive likelihood ratio of 8.3 for a diagnosis of CS. In the setting of known extracardiac sarcoidosis, specificity was 97%, PPV 94% and positive likelihood ratio 11.9. However, this echocardiographic model had limited sensitivity of 33% and 34% among the whole group and the extracardiac sarcoidosis patients, respectively. Indeed, all three echocardiographic models had limited ‘rule-out’ ability as evidenced by the modest NPV and negative likelihood ratios.

**Table 7:** Diagnostic ability of TTE graded by likeliness to predict CS among (a) all patients and (b) those with proven extracardiac sarcoidosis

TTE Model	Abnormal TTE (*n*, %)	Sens (95% CI)	Spec (95% CI)	PPV (95% CI)	NPV (95% CI)	LR+ (95% CI)	LR- (95% CI)
		Whole group (*n* = 181)					
Probable CS	38	33 (24–43)	96 (89–99)	92 (79–98)	50 (42–59)	8.3 (2.6–25.9)	0.7 (0.6–0.8)
Highly likely CS	59	48 (38–58)	89 (90–95)	86 (75–94)	55 (46–64)	4.5 (2.3–8.9)	0.6 (0.5–0.7)
Moderately likely CS	79	54 (44–64)	71 (59–81)	72 (61–82)	52 (42–62)	1.8 (1.2–2.7)	0.7 (0.5–0.8)
		In the setting of extracardiac sarcoidosis (*n* = 167)					
Probable CS	35	34 (25–44)	97 (90–100)	94 (81–99)	52 (43–60)	11.9 (2.9–48.0)	0.7 (0.6–0.8)
Highly likely CS	52	46 (36–57)	90 (81–96)	87 (74–94)	55 (45–64)	4.6 (2.2–9.7)	0.6 (0.5–0.7)
Moderately likely CS	72	53 (42–63)	70 (58–80)	71 (59–81)	52 (41–62)	1.8 (1.2–2.6)	0.7 (0.5–0.9)

### Diagnostic tests for cardiac involvement among patients with known extracardiac sarcoidosis

Among the 167 patients with extracardiac sarcoidosis, a significantly higher proportion of those with cardiac involvement had an abnormal ECG (45 [46%] vs. 8 [12%], *p* < 0.001), Holter monitor (33 [34%] vs. 7 [10%], *p* < 0.001) and probable TTE (33 [34%] vs. 2 [3%]), *p* < 0.001) compared to those without cardiac involvement. There was no significant difference in proportion of patients presenting with chest pain (*p* = 0.478) or palpitations (*p* = 0.151). However, CS+ patients were more likely to present with pre-syncope or syncope (21 [22%] vs. 7 [10%], *p* = 0.047).

**Table 8:** Ability of investigations to detect cardiac involvement among symptomatic and asymptomatic sarcoidosis patients

	Sens, %(95% CI)	Spec, %(95% CI)	PPV(95% CI)	NPV(95% CI)	LR+(95% CI)	LR-(95% CI)
**Existing screening criteria**						
2014 HRS	77 (68–85)	37 (26–50)	63 (54–72)	54 (39–69)	1.23 (1.00-1.52)	0.61 (0.38–0.98)
2020 ATS	84 (75–90)	26 (16–38)	61 (52–69)	53 (35–70)	1.12 (0.95–1.32)	0.64 (0.35–1.17)
**Positive abnormal test**:						
Cardiac symptoms	73 (63–82)	30 (20–42)	59 (50–68)	45 (30–60)	1.05 (0.86–1.27)	0.89 (0.55–1.45)
ECG	46 (36–57)	88 (78–95)	85 (72–93)	54 (44–63)	4.00 (2.02–7.94)	0.61 (0.49–0.74)
Holter monitor	34 (25–44)	90 (80–96)	83 (67–93)	49 (40–58)	3.35 (1.58–7.13)	0.73 (0.62–0.86)
Any rhythm (ECG or Holter)	61 (50–71)	86 (75–93)	86 (75–93)	61 (51–71)	4.26 (2.35–7.72)	0.46 (0.35–0.60)
‘Probable CS’ TTE	34 (25–44)	97 (90–100)	94 (81–99)	52 (43–60)	11.91 (2.95–47.99)	0.68 (0.58–0.79)
CMR	99 (94–100)	79 (67–88)	87 (79–93)	98 (90–100)	4.59 (2.89–7.31)	0.01 (0.00-0.09)
FDG-PET alone	47 (37–58)	99 (92–100)	98 (89–100)	58 (48–67)	33.20 (4.69–235.00)	0.53 (0.44–0.65)
FDG & Rb-82-PET combined	68 (58–77)	94 (86–98)	94 (86–98)	68 (58–77)	11.91 (4.55–31.14)	0.34 (0.25–0.46)
**Screening combination**:						
Any of symptoms, ECG, Holter, TTE	90 (82–95%)	26 (16–38%)	63 (54–71)	64 (44–81)	1.21 (1.04–1.41)	0.40 (0.20–0.81)
**Symptomatic**:						
Ab rhythm + Ab TTE	17 (10–25)	100 (95–100)	100 (79–100)	46 (38–55)	∞	0.84 (0.76–0.91)
Ab rhythm + Normal TTE	33 (23–43)	90 (81–96)	82 (67–93)	49 (40–58)	3.30 (1.55–7.04)	0.74 (0.63–0.87)
Normal rhythm + Ab TTE	3 (1–9)	99 (92–100)	75 (19–99)	42 (35–50)	2.16 (0.23–20.38)	0.98 (0.94–1.03)
Normal rhythm + Normal TTE	21 (13–30)	41 (30–54)	33 (21–46)	27 (19–37)	0.35 (0.23–0.55)	1.92 (1.42–2.58)
**Asymptomatic**:						
Ab rhythm + Ab TTE	9 (4–17)	99 (92–100)	90 (56–100)	44 (36–52)	6.49 (0.84–50.10)	0.92 (0.86–0.99)
Ab rhythm + Normal TTE	2 (0–7)	97 (90–100)	50 (7–93)	42 (34–50)	0.72 (0.10-5.00)	1.01 (0.96–1.06)
Normal rhythm + Ab TTE	5 (2–12)	100 (95–100)	100 (48–100)	43 (36–51)	∞	0.95 (0.91–0.99)
Normal rhythm + Normal TTE	10 (5–18)	76 (64–85)	37 (19–58)	38 (30–46)	0.42 (0.21–0.87)	1.18 (1.02–1.37)

### Diagnostic ability of cardiac tests in screening for cardiac involvement among extracardiac sarcoidosis patients

The diagnostic ability for each test in isolation and in combination to detect CS is detailed in Table 8. As expected, CMR had the highest sensitivity (99%) for the detection of CS as a stand-alone investigation and had excellent ‘rule-out’ ability (NPV or 98% and negative likelihood ratio of 0.01). Individually, symptom evaluation, 12-lead ECG, Holter monitoring and TTE had modest sensitivity. However, when the data was combined the sensitivity was 90%, negative likelihood ratio 0.40 and NPV 64%. This approach carried greater sensitivity, NPV and lower negative likelihood ratio than the 2020 American Thoracic Society (Sens 84%, NPV 53%, negative likelihood ratio 0.64) and 2014 h screening criteria (Sens 77%, NPV 54%, negative likelihood ratio 0.61).

**Fig. 3:**
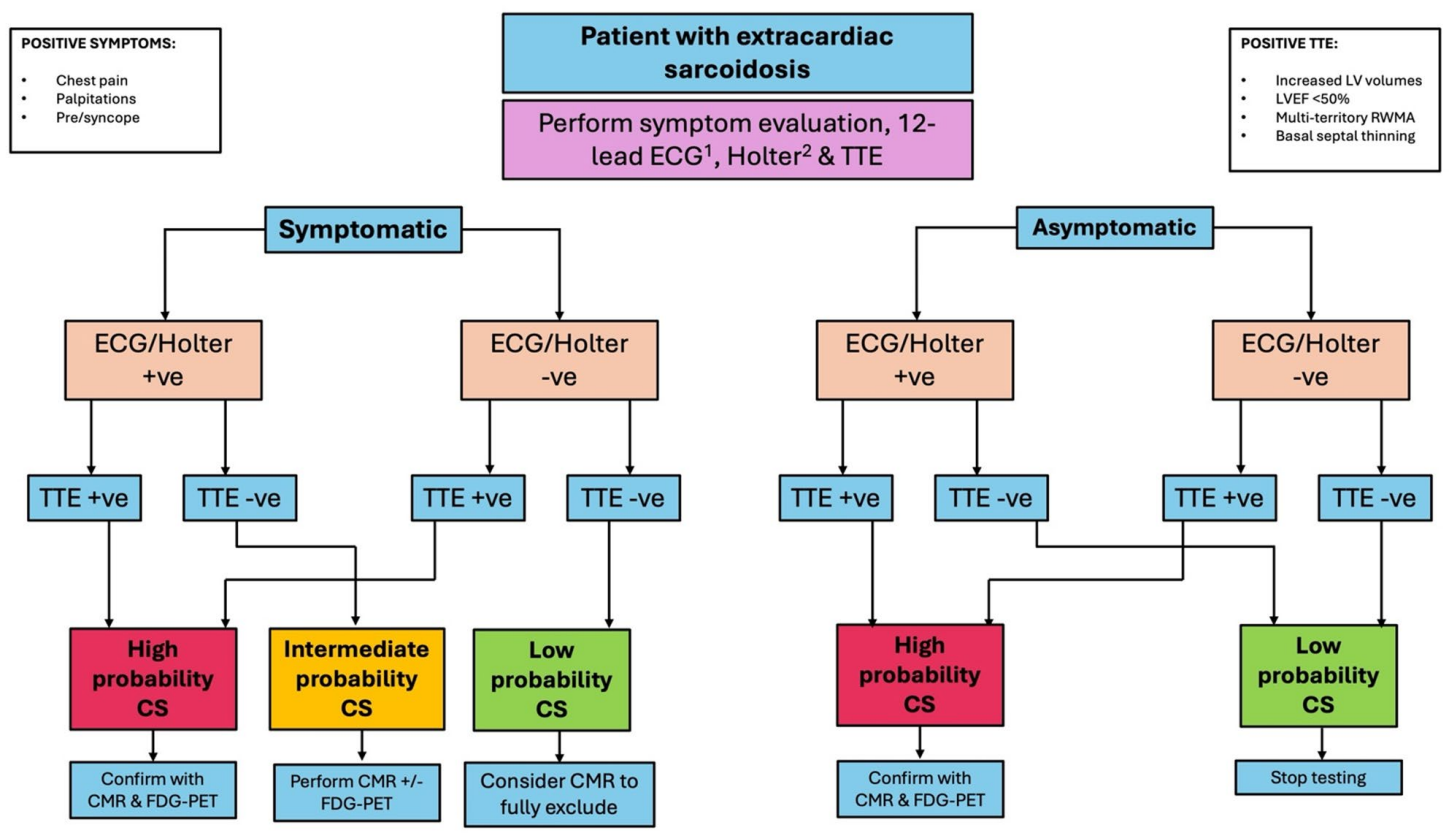
Recommended pathway for screening of sarcoidosis patients for cardiac involvement. In sarcoidosis patients it is recommended to first perform detailed history taking for the presence of cardiac-sounding symptoms (chest pain, palpitations, pre-syncope or syncope). Then onward performance of a12-lead electrocardiogram (ECG), ambulatory Holter monitoring for rhythm data and a transthoracic echocardiogram (TTE). In the symptomatic patient, regardless of the findings on ECG/Holter, an abnormal TTE suggests a high probability of CS (positive predictive value of 99–100%) which should be confirmed with CMR and FDG-PET. If the TTE is normal, the presence of abnormal ECG/Holter suggests an intermediate probability of CS (positive predictive value of 82%) and CMR should be performed (followed by FDG-PET if the CMR is positive). Normal rhythm data and TTE supports a low probability of CS. While 12% of patients will still have cardiac involvement, the lack of TTE findings indicates a low-risk cohort. Depending on clinical preference, CMR may be performed to fully exclude CS. In the asymptomatic patient, regardless of the findings on ECG/Holter, an abnormal TTE suggests a high probability of CS (positive predictive value of 90–100%) which should be confirmed with CMR and FDG-PET. Only a minority (1%) of asymptomatic patients displayed abnormal rhythm data with a normal TTE. The presence of both normal rhythm data and TTE supports a low probability of CS with only 6% of patients likely to cardiac involvement in what is a low-risk cohort. ^1^Abnormal ECG = presence of left or right bundle branch block, pathological Q-waves, sustained or non-sustained ventricular tachycardia (VT), ventricular ectopy any degree of atrioventricular (AV) block or supraventricular arrhythmia. ^2^Abnormal Holter = presence of ventricular ectopic burden > 10%, supraventricular ectopic burden > 10%, NSVT, sustained VT, supraventricular tachycardia, high degree AV block or sinus node dysfunction over 24 h, 48 h or 7-day ambulatory monitoring

In the symptomatic patient with extracardiac sarcoidosis, the combination of any abnormal rhythm data (ECG or Holter) plus an abnormal TTE had 100% PPV with 100% specificity for a CS diagnosis. This was reduced to 90% PPV and 99% specificity in the absence of cardiac-sounding symptoms. Conversely, both normal rhythm data and normal TTE made CS unlikely but did not fully exclude it. In the symptomatic patient with extracardiac sarcoidosis, even in the setting of normal data and TTE, 12% of patients went on to be diagnosed with CS. This fell to 6% in the absence of symptoms. Accordingly, Fig. 3 depicts our proposed screening strategy.

## Discussion

This was a large prospective study evaluating the ability of echocardiography to diagnose CS, incorporating both strain and 3D imaging, over and above conventional echocardiography. The most specific echocardiographic markers of CS were LV cavity dilatation, RWMA presence in multiple myocardial territories, reduced LVEF < 50% and basal septal thinning. The addition of strain failed to improve the overall diagnostic accuracy. When combined with symptoms and rhythm data, echocardiography remains a useful screening tool. Here, we propose a novel approach in the screening of sarcoidosis patients for cardiac involvement.

### The role of echocardiography in the diagnosis of CS

The 2014 HRS criteria recognize that RWMA, scarring or aneurysm formation on TTE should prompt the need for further imaging investigations, but do not consider these findings sufficient for a CS diagnosis [4]. Indeed, the only *diagnostic* criterion that can be fulfilled by echocardiography is an unexplained reduction in LVEF to < 40%. Since the majority of CS patients have preserved LVEF at the time of diagnosis this, in part, explains the low sensitivity of conventional echocardiography for diagnosing CS. The recommendations within the HRS criteria were based on 2 retrospective studies between 2008 and 2013 [6, 30]. Mehta et al. studied 62 patients and showed that an abnormal TTE had 25% sensitivity and 95% specificity to detect CS, with a positive and negative likelihood ratio of 4.8 and 0.8, respectively [6]. An abnormal TTE was defined as LVEF ≤ 45% and/or > 2 segments of RWMA and/or RV systolic impairment in the absence of pulmonary hypertension and/or significant diastolic dysfunction. Freeman et al. found that among 70 patients, the presence of any major (reduced LVEF) or minor (RV systolic impairment, RWMA, wall thinning or diastolic dysfunction) TTE findings had 62% sensitivity and 29% specificity for detecting CS [30]. More contemporaneous studies have reported a sensitivity of 27–32%, which is identical to the 33% identified in our present cohort [7, 8]. Our findings indicate that the TTE parameters most specific for CS were LV cavity dilatation, reduced LVEF < 50%, multi-territory RMWA presence and basal septal thinning. In the setting of extracardiac sarcoidosis, the presence of one more of these findings was associated with a 94% PPV for the disease. The strong association reported between LV cavity dilatation and cardiac involvement, independent of impaired LVEF, is novel compared to prior studies and may represent the development of dilatation before decline in systolic function. Previous work by our group has identified that in almost 40% of CS patients undergoing multimodality imaging (TTE, CMR & FDG-PET) at the time of diagnosis, TTE with RWMA could identify those at heightened risk of adverse cardiac risk [3]. The additional data provided by our current study shows that these patients can identified at the screening stage with reliable accuracy. This lends added weight to the argument for performing TTE during the screening phase. We found that 3DE data did not improve the overall diagnostic yield from echocardiography, hampered by feasibility of only 50% for accurate 3D imaging.

### The role of strain in detecting CS

Previous studies have identified GLS and GCS to be significantly more impaired among sarcoidosis patients without cardiac involvement compared to healthy controls [10–15, 31]. However, few have attempted to evaluate the ability of strain to diagnose cardiac involvement within a suspected CS cohort. Circumferential layer performance is highly sensitive to injury within midwall myocardial fibres [32]. Consequently, the presence of midwall fibrosis tends to be associated with impairment of GCS in non-ischaemic cardiomyopathies [33]. In CS, the predominant pattern of fibrosis seen on CMR is epicardial and/or midwall, with a predilection for the septum [34, 35]. Kusunose et al. found that longitudinal strain in the basal segments was significantly more impaired among 18 CS+ compared to 82 CS- patients [13]. Jankowska et al. failed to detect any significant difference in LVGLS between 43 CS + and 76 CS- patients but did find partial correlation with impaired segmental longitudinal strain values and presence of LGE on CMR [16]. These findings contrasted with those of Murtagh et al. who found LVGLS to be more reduced in 31 CS+ compared to 31 CS- patients with LVEF > 50% [17]. An LVGLS cutoff of -17% showed 94% sensitivity and 94% specificity for a CS diagnosis.

In our study we found an LVGLS cutoff of -13% and LVGCS of -15% had ≥ 90% specificity for CS. Importantly these cutoffs are significantly more abnormal that those in the referenced normal range which could make it easier for clinicians to identify pathological states. By incorporating the strain parameters, we improved the sensitivity of TTE from 33% to 54%.

However, this resulted in a reduction in specificity from 96% to 71% and positive likelihood ratio from 8.3 to 1.8, thus failing to improve the overall diagnostic ability of this modality. Interestingly, significant differences in 3D strain values were not observed in those with and without CS. This is most likely explained by the reduced number of feasible studies and lower reproducibility of 3D strain measurements.

### The role of echocardiography in screening for cardiac involvement among sarcoidosis patients

In CS, the optimal screening strategy should prioritize less false negatives over less false positives. As the clinical algorithm recommends onward performance of CMR and FDG-PET following an abnormal echocardiogram, it is important not to miss patients who may benefit from more definitive investigation. Therefore, the optimal screening approach requires a high sensitivity for the detection of disease. In our study, this involved integrating symptoms, 12-lead ECG (or ambulatory Holter) and TTE, to achieve a sensitivity of 90%. We recommend using the TTE model with the greatest likelihood of detecting CS in order to balance the relatively low specificity provided by the other screening tests.

### Comparison between our screening strategy and current guidelines

Within our cohort, the integrated approach appeared to outperform both the HRS and ATS screening criteria, which had sensitivities of 77% and 84%, respectively. Our screening algorithm differs from the HRS screening criteria in several ways. Firstly, by highlighting the relative significance of positive rhythm and TTE data depending on the presence or absence of cardiac-sounding symptoms. Secondly, we included chest pain within the symptom screen. Thirdly, our wider definition of a positive TTE contrasts with that within the HRS criteria whereby the presence of a RWMA and/or LVEF < 40% were deemed abnormal features [4]. In our case the LVEF cutoff of 50%, better reflects the current definition of LV impairment. The additional use of LV cavity dilatation and highly specific RWMA findings reflects a more precise TTE phenotype. The ATS screening criteria differs to the HRS in that the authors do not recommend TTE or ambulatory ECG monitoring for screening, albeit acknowledging the lack of evidence for this recommendation [9]. The ATS statement recommends selective use of CMR in sarcoidosis patients, based on symptomatic screening. Due to the high prevalence of subclinical CS, a low threshold for CMR is emphasised. However, routine screening of sarcoidosis patients with CMR has cost implications, variable accessibility, and is yet to be validated. The incorporation of echocardiography allows for rapid bedside assessment of biventricular function and pulmonary artery pressure, important in the clinical management of suspected CS [5]. Our study adds to the available literature by highlighting an increase in sensitivity from 84% using only symptoms and ECG, to 90% using TTE. Our findings therefore support the integration of TTE within a screening algorithm for sarcoidosis patients. However, CMR remains the key rule-out modality and should be performed if any clinical uncertainty remains.

### Limitations

This was a single-centre study and therefore referral bias is likely. In particular, as a national sarcoidosis unit, a higher rate of CS diagnosis may have been observed compared to other centres. While echocardiograms were performed by more than one operator, all echocardiographers were highly experienced and all final measurements were performed and analysed by one investigator. As only 50% of scans were feasible for 3D analysis this may have resulted in a relative bias against the ability of 3DE in detecting CS. Evaluation of 2D LVGRS was not performed, however no significant findings were observed with the use of 3D LVGRS. Due to inter-vendor differences in strain values dependent on the software used, caution must be applied when extrapolating data performed on alternative software.

## Conclusions

Echocardiography serves as a useful screening tool for the detection of LV systolic dysfunction, cavity dilatation or RWMA in identifying patients with suspected CS who may benefit from further detailed imaging investigation. The echocardiographic detection of cardiac involvement is modestly enhanced by the addition of strain. Contemporary advanced echocardiography lacks the overall discriminatory ability to be a standalone diagnostic test but has clinical value in combination with symptom evaluation and rhythm data. Onward performance of CMR and FDG-PET imaging remain key investigations in the detection and management of CS.

## Supplementary Information

Below is the link to the electronic supplementary material.

Supplementary Material 1

## Data Availability

The datasets used and/or analysed during the current study are available from the corresponding author on reasonable request.
