# Supplementary material for: Evaluation of contemporary echocardiography for the detection of cardiac sarcoidosis
Source: Echo Res Pract. 2026 Apr 27;13:12. doi: 10.1186/s44156-026-00111-9 (PMC13112891; doi:10.1186/s44156-026-00111-9)
Supplement: Supplementary file 1 — Supplementary Material 1 [file 44156_2026_111_MOESM1_ESM.docx]

**Supplemental files**

**Supplemental 1: Cardiac sarcoidosis diagnostic criteria**

## Heart Rhythm Society 2014 criteria

The 2014 HRS consensus statement allows a ‘definite’ histological diagnosis to be made from endomyocardial biopsy demonstrating non-caseating granulomas. A clinical CS diagnosis is ‘probable’ in the presence of a histological diagnosis of extracardiac sarcoidosis (e.g. endobronchial ultrasound-guided biopsy for pulmonary sarcoidosis) plus one of the following:

- Steroid +/- immunosuppressant responsive cardiomyopathy or heart block
- Unexplained reduced LVEF (<40%)
- Unexplained sustained (spontaneous or induced) VT
- Mobitz type II 2^nd^ degree heart block or 3^rd^ degree heart block
- Patchy uptake on dedicated cardiac PET in a pattern consistent with CS
- Late gadolinium enhancement on CMR in a pattern consistent with CS
- Positive gallium uptake in a pattern consistent with CS

Other causes for the above cardiac manifestations must be reasonably excluded.

## Japanese Circulation Society 2016 criteria

In 2016 the Japanese Circulation Society (JCS) published update guidance on the diagnosis of CS (Terasaki JCS). The main divergence from the HRS 2014 statement included the ability to diagnose extracardiac sarcoidosis with and without histology, thus reflecting trends in clinical practice. In the absence of histology, a diagnosis of extracardiac sarcoidosis may be established if at least two of the five characteristics are observed:

1. Bilateral hilar lymphadenopathy
2. High serum angiotensin-converting enzyme (ACE) activity of elevated serum lysozyme levels
3. High serum soluble interleukin-2 receptor (sIL-2R) levels
4. Significant tracer accumulation in ^67^Ga citrate scintigraphy or ^18^F-FDG-PET
5. A high percentage of lymphocytes with s CD4/CD8 ratio of >3.5 in bronchoalveolar lavage fluid

Similar to the HRS 2014 guidance, CS may be diagnosed either histologically with positive endomyocardial biopsy, or clinically. A clinical diagnosis is established if ≥2 major criteria are satisfied or one major plus ≥2 minor criteria are satisfied:

1. Major criteria

(a) High-grade AV block or fatal VA (sustained VT/VF)

(b) Basal thinning of the ventricular septum or abnormal ventricular wall anatomy (ventricular aneurysm, thinning of the middle or upper ventricular septum, regional ventricular wall thickening)

(c) Left ventricular contractile dysfunction (LVEF <50%) or focal ventricular wall asynergy

(d) ^67^Ga citrate scintigraphy or ^18^F-FDG PET reveals abnormally high tracer accumulation in the myocardium

(e) LGE CMR reveals delayed contrast enhancement of the myocardium

2. Minor criteria

(f) Abnormal ECG findings: Ventricular arrhythmias (non-sustained VT, multifocal or frequent premature ventricular contractions), bundle branch block, axis deviation, or abnormal Q waves

(g) Perfusion defects on myocardial perfusion scintigraphy (SPECT)

(h) Endomyocardial biopsy: Monocyte infiltration and moderate or severe myocardial interstitial fibrosis

## Japanese Circulation Society 2016 – isolated cardiac sarcoidosis criteria

The 2016 JCS criteria is the first to provide guidance on diagnosing isolated. In principle, when the presentation of CS is highly suggestive, but disease involves only the heart and cannot be detected in other organs despite extensive extra-cardiac investigation, isolated cardiac sarcoidosis is likely. Thorough clinical examination and investigation of the pulmonary, ophthalmic, musculoskeletal and skin organs to exclude the presence of extracardiac sarcoidosis are essential. The presence of other myocardial diseases such as coronary artery disease and myocarditis should also be ruled out. Whole body FDG-PET imaging is particularly useful in determining whether extra-cardiac inflammation may be present. A prerequisite for diagnosis includes:

1. No clinical findings characteristics of sarcoidosis are observed in any organs other than the heart. The patient should be examined in detail for respiratory, ophthalmic, and skin involvements of sarcoidosis. When the patient is symptomatic, other aetiologies that can affect the corresponding organs must be ruled out.
2. ^67^Ga scintigraphy or ^18^F-FDG PET reveals no abnormal tracer accumulation in any organs other than the heart.
3. A chest CT scan reveals no shadow along the lymphatic tracts in the lungs or no hilar and mediastinal lymphadenopathy (minor axis >10 mm).

Once all three have been fulfilled a histological diagnosis is fulfilled with positive endomyocardial biopsy. A clinical diagnosis is fulfilled once criterion (d) *“^67^Ga citrate scintigraphy or ^18^F-FDG PET reveals abnormally high tracer accumulation in the myocardium”* plus at least 3 of the other major criteria (a) to (e) are present.

| **Supplemental 2: Table of recommended screening strategy from 2014 HRS and 2020 ATS guidelines** | | |
| --- | --- | --- |
|  | **Heart Rhythm Society algorithm** | **American Thoracic Society algorithm**  **(asymptomatic)** |
| **Cardiac symptoms** | - Palpitations - Presyncope/syncope | - Palpitations - Presyncope/syncope - Chest pain |
| **12-lead ECG** | - Left or right bundle branch block - Pathological Q-waves - Sustained or non-sustained ventricular tachycardia - 2^nd^ or 3^rd^ degree atrioventricular block | ***Not specified so our criteria used:***   - Left or right bundle branch block - Pathological Q-waves - Sustained or non-sustained ventricular tachycardia - Ventricular ectopy - Any degree of atrioventricular block - Any supraventricular arrhythmia |
| **Ambulatory Holter monitoring** | - Not recommended | - Not recommended |
| **Conventional transthoracic echocardiogram** | - RWMA - Wall aneurysm - Basal septal thinning - LVEF <40% | - Not recommended |

## Supplemental 3: FDG-PET and CMR protocols

FDG-PET protocol

Our FDG and Rubidium-82 (Rb-82) PET protocol has been previously described [1]. In summary, images were acquired using the Siemens mCT flow system (Siemens Healthcare, Germany). A specialist blinded to patient data analysed attenuation-corrected inflammatory using MIMCardiac (MIM Software Inc, Cleveland, Ohio) and perfusion PET images using QGS+QPS (Cedars-Sinai Medical Centre). Focal or focal-on-diffuse FDG uptake was considered compatible with active CS. Diffuse FDG uptake involving the whole myocardium in the absence of corresponding perfusion defect on Rb-82 PET was deemed a diagnostic failure due to poor suppression of physiologic glucose uptake and excluded from the final analysis. The maximum standardized uptake value (SUVmax) within the heart was determined automatically. Active inflammation was defined as the presence of focal or focal-on-diffuse FDG uptake with SUVmax ≥2.5. An abnormal FDG/Rb-82-PET was defined as the presence of active inflammation +/- perfusion defect compatible with CS

CMR protocol

All CMR scans were performed on a 1.5-Tesla system (Magnetom Sonata, Avanto, or Aera; Siemens, Erlangen, Germany). Imaging protocols included steady-state free precession (SSFP) breath-hold cines for the assessment of ventricular volumes, function and morphology, and LGE sequences for the detection of myocardial fibrosis, as per recommendations [2]. Additional tissue characterization images of T2-weighted imaging were added to the protocol.

CMR analyses were performed by an expert reader blinded to all other patient information using semi-automated software (CMR tools; Cardiovascular Imaging Solutions, London). Ventricular volumes and ejection fraction were quantified by planimetry of end-diastolic and end-systolic endocardial borders on short-axis cine images from apex to base. LGE presence and location was identified visually in both ventricles. An abnormal CMR was defined as the presence of an LGE pattern and distribution compatible with a probable or highly probable CS diagnosis [3].

References:

1. Okafor J, Khattar R, Kouranos V, et al. Role of serial ^18^F-fludeoxyglucose positron emission tomography in determining the therapeutic efficacy of immunosuppression and clinical outcome in patients with cardiac sarcoidosis. *J Nucl Cardiol*. 2024;35:101842. doi:10.1016/j.nuclcard.2024.101842
2. Kramer CM, Barkhausen J, Bucciarelli-Ducci C, Flamm SD, Kim RJ, Nagel E. Standardized cardiovascular magnetic resonance imaging (CMR) protocols: 2020 update. *J Cardiovasc Magn Reson*. 2020;22(1):17. doi:10.1186/s12968-020-00607-1
3. Vita T, Okada DR, Veillet-Chowdhury M, et al. Complementary Value of Cardiac Magnetic Resonance Imaging and Positron Emission Tomography/Computed Tomography in the Assessment of Cardiac Sarcoidosis. *Circ Cardiovasc Imaging*. 2018;11(1):e007030. doi:10.1161/CIRCIMAGING.117.007030

| **Supplementary 4: Table of two-way mixed-effects model intra-observer intraclass correlations for LV speckle-tracking echocardiography (n=30 patients)** | | |
| --- | --- | --- |
|  | **ICC** | **95% CI** |
| 2D GLS | 0.911 | 0.781-0.962 |
| 2D GCS | 0.960 | 0.903-0.981 |
| 3D GLS | 0.763 | 0.559-0.879 |
| 3D GCS | 0.746 | 0.532-0.871 |
| 3D GRS | 0.569 | 0.269-0.769 |
| 3D GPS | 0.650 | 0.383-0.816 |
| 3D Twist | 0.718 | 0.486-0.854 |
| 3D Torsion | 0.701 | 0.461-0.848 |
